# Biochemical and Bioinformatic Characterization of Type II Metacaspase Protein (TaeMCAII) from Wheat

**DOI:** 10.1007/s11105-012-0450-6

**Published:** 2012-04-20

**Authors:** E. Piszczek, M. Dudkiewicz, M. Mielecki

**Affiliations:** 1 Department of Biochemistry, Warsaw University of Life Sciences, Nowoursynowska 159, 02776 Warsaw, Poland; 2 Department of Experimental Design and Bioinformatics, Warsaw University of Life Sciences, Nowoursynowska 159, 02776 Warsaw, Poland; 3 Department of Protein Biosynthesis Institute of Biochemistry and Biophysics, Polish Academy of Sciences, Pawinskiego 5a, 02106 Warsaw, Poland

**Keywords:** Type II wheat metacaspase, Cysteine-dependent autoprocessing, Calcium ions, Programmed cell death

## Abstract

The biochemical analysis and homology modeling of a tertiary structure of a cereal type II metacaspase protein from wheat (*Triticum aestivum*), TaeMCAII, are presented. The biochemical characterization of synthetic oligopeptides and protease inhibitors of *Escherichia coli*-produced and purified recombinant TaeMCAII revealed that this metacaspase protein, similar to other known plant metacaspases, is an arginine/lysine-specific cysteine protease. Thus, a model of a plant type II metacaspase structure based on newly identified putative metacaspase-like template was proposed. Homology modeling of the TaeMCAII active site tertiary structure showed two cysteine residues, Cys140 and 23, in close proximity to the catalytic histidine, most likely participating in proton exchange during the catalytic process. The autoprocessing that leads to activation of TaeMCAII was highly dependent on Cys140. TaeMCAII required high levels of calcium ions for activity, which could indicate its involvement in stress signaling pathways connected to programmed cell death.

## Introduction

Programmed cell death (PCD) is a process of elimination of unwanted cells during the ontogenesis of organisms and in response to environmental stresses. It is common to all eukaryotic cells, including animal and plant cells. Plants and animals share many similarities in the morphological features and enzymatic machinery of PCD (Sun et al. 2012; Sanmartin et al. 2005). The initiators and executors of animal PCD are caspases, a family of cysteine-dependent proteases that cleave their substrates at the carboxyl-terminal side of aspartate residues. They are synthesized as inactive proenzymes that comprise an N-terminal prodomain together with one large and one small subunit. The crystal structures of caspases show that the active enzymes are heterotetramers that contain two small and two large subunits. The enzymes have two active sites that are found at opposite ends of the molecules. Both the small and large subunits participate in the formation of active site. Two residues, cysteine and histidine, are present in the active sites and participate in catalysis (Ho and Hawkins 2005; Cohen 1997). The activation of caspases during PCD processes such as apoptosis and autophagy results in the cleavage of important cellular proteins, including poly(ADP-ribose) polymerase and lamins, leading to the demise of the cell (Earnshaw et al. 1999).

The existence of distant caspase relatives named caspase-like proteases has been demonstrated previously in plant cells undergoing PCD (Sanmartin et al. 2005). Metacaspases, which are caspase-like enzymes, were discovered in silico in the *Arabidopsis* genome more than a decade ago, and they are present in protozoa, fungi and plants (Uren et al. 2000). Phylogenetic analysis has revealed that metacaspases are distant homologs and ancestors of animal caspases (Vercammen et al. 2007). These proteases, together with eukaryotic caspases, metazoan paracaspases, legumains, separases and the bacterial clostripains and gingipains, are classified as members of clan CD cysteine proteases (Bonneau et al. 2008). All proteins from this clan share a common structural feature, the presence of the caspase–hemoglobinase fold (Bonneau et al. 2008).

On the basis of metacaspase structure, they can be subdivided into two groups: type I and type II. Type I metacaspases possess an N-terminal prodomain with a Zn finger motif, which is absent in type II. The distinguishable feature of type II metacaspases is the presence of a linker region between the large and the small subunit (Piszczek and Gutman 2007). Until now, it has been shown for *Arabidopsis thaliana* and *Picea abies* type II metacaspases that they are synthesized as inactive zymogens, and that they are activated by autoprocessing, similar to effector caspases from mammals (Vercammen et al. 2004; Bozhkov et al. 2005). In contrast, type I metacaspases from *Arabidopsis* do not autoprocess, and most likely similar to initiator mammalian caspases, they require oligomerization for activity (Vercammen et al. 2004). Metacaspases and animal caspases contain a conserved catalytic His/Cys dyad in their active site, with the Cys residue acting as a nucleophile for substrate peptide bond hydrolysis (Piszczek and Gutman 2007). The striking difference between all discovered metacaspases and caspases is the formers’ preference for Arg or Lys residues in their substrates (Vercammen et al. 2004; Bozhkov et al. 2005). Metacaspase activities can be modified by post-translational modification and through the action of inhibitors. The zymogen of metacaspase 9 of *A. thaliana* (AtMC9), but not its active form, is modified post-translationally by S-nitrosylation (Belenghi et al. 2007). The same modification can also influence caspase activities (Lai et al. 2011). Furthermore, the activity of AtMC9 can be inhibited by serpin-1, an inhibitor of serine proteases (Vercammen et al. 2006). Apart from serine proteases, some serpins also inhibit caspase-1, 8 and 10 (Ye and Goldsmith 2001). Because of the mode of its inhibitory action on AtMC9, serpin-1 is also the first identified natural metacaspase substrate (Vercammen et al. 2006). Recently, an animal caspase-3 substrate, tudor staphylococcal nuclease (TSN), was discovered to be a natural substrate of *Picea abies* metacaspase mcII-Pa during both developmental and stress-induced programmed cell death (PCD) (Sunström et al. 2009).

Similar to caspases, metacaspases are multifunctional proteins that take part in the regulation and the execution of PCD, cell cycle control, aging and oxidative stress (Tsiatsiani et al 2011). A striking example of a multifunctional metacaspase is YCA1 from budding yeast (*Saccharomyces cerevisiae*), which was found to be a positive regulator and active player in oxidative stress and senescence-associated PCD in addition to being a cell cycle controller (Madeo et al. 2004). Moreover, the participation of YCA1 in the clearance of insoluble protein aggregates during physiological and stress conditions provides a vital non-death cell function for metacaspases (Lee et al. 2010). Another metacaspase, mcII-Pa, is involved in developmental PCD during the early stages of embryogenesis from the gymnosperm plant Norway spruce (*Picea abies*) (Bozhkov et al. 2005). Interestingly, *AtMC8* metacaspase from *A. thaliana*, was found to be upregulated during oxidative stress PCD pathways that were induced by UV-C, H_2_O_2_ and methyl viologen (He et al. 2008).

To date, very few plant metacaspases have been identified and well characterized. Most biochemical and functional findings described so far come from *Arabidopsis* and *Picea abies* studies (Vercammen et al. 2004; Bozhkov et al. 2005). Here, we provide a biochemical analysis of the cereal metacaspase from *Triticum aestivum*, TaeMCAII, including molecular modeling of its tertiary structure. *TaeMCAII* cDNA encodes a protein of 405 amino acids with a molecular mass of 44 kDa and an isoelectric point of 5.29 (Piszczek et al. 2011).

## Materials and Methods

### Plants and Growth Conditions

Spring wheat (*Triticum aestivum* L.) seedlings were grown in Hoagland nutrient solution for 2 weeks under controlled conditions (temperature of 20°C, 16 h photoperiod, irradiance of 260 μmol m^−2^ s^−1^ and a relative humidity of 70–80 %). The plants were subjected to heat shock at a temperature of 50°C for 20 min to evoke PCD. Wheat leaves were collected 3 h after stress and were frozen in liquid nitrogen and stored at −80°C until the isolation of total RNA according to the method of Chomczyński and Sacchi (1987).

### Cloning of Type II Metacaspase Open Reading Frame from *Triticum aestivum*

cDNA was synthesized using a reverse transcriptase system (Promega, Madison, WI) from an RNA template obtained from 2-week-old spring wheat seedlings (*Triticum aestivum* L.) that were exposed to heat shock (50 °C) for 20 min. The cDNA and forward and reverse primers with sequences of 5′-CGCAACATTGGATCCATGGGCCGCAAGCTCGCGCTCCTGGTGGGCATC-3′ and 5′-GATATTGATCTCGAGTCAGCAGATGAAAGCCACATGAACATGCTCATC-3′, respectively, were used for the PCR reaction using the KOD Hot Start polymerase (Novagen). The PCR reaction was performed under the following conditions: 95°C at 2 min, 35 cycles of 20 s at 95°C, 10 s at 60°C and 30 s at 70°C. The PCR product was cloned into the bacterial expression vector pET-28a(+) (Novagen), which resulted in an N-terminal fusion with an His_6_ epitope tag.

### Production of TaeMCAII in *Escherichia coli*

A plasmid vector harboring the open reading frame of TaeMCAII was transformed into *Escherichia coli* strain BL21(DE3)pLysS. Bacterial cultures were grown in LB medium supplemented with 50 μg ml^−1^ kanamycin and 50 μg ml^−1^ chloramphenicol at 37°C until the culture reached an optical density of 0.6–0.8 at 600 nm. Protein expression was induced by the addition of 1 mM isopropyl-thiogalactoside (IPTG) for 3 h. The bacteria were centrifuged at 5,000 rpm at 4°C for 15 min. The pellet was resuspended in protein extraction buffer that contained 20 mM Tris-HCl (pH 8.0), 0.5 M NaCl, 5 % glycerol, 0.1 % Triton X, 5 mM β-mercaptoethanol and protease inhibitor Complete (Roche, Mannheim, Germany) and was then sonicated. The bacterial lysate was centrifuged at 20,000 rpm for 30 min at 4°C, and the supernatant was mixed with 1 ml Ni-NTA agarose resin (Invitrogen, La Jolla, CA) for 1 h with the addition of 10 mM imidazole to bind the recombinant protein. The Ni-NTA agarose column containing bound protein was then washed with protein extraction buffer supplemented with 20 mM imidazole five times. The protein was eluted with the same buffer containing 300 mM imidazole and dialyzed overnight against MTSB buffer containing 20 mM PIPES (pH 7.0), 2 mM EGTA, 2 mM MgSO_4_, 10 % glycerol, 50 mM NaCl and 2 mM DTT.

### Cloning and Expression of TaeMCAII Cys140 and Cys23 Mutants

To investigate the involvement of the two cysteine residues that make up the active site of TaeMCAII, two mutant forms, TaeMCAIIC140A and TaeMCAIIC23A, were cloned and expressed in *E. coli* strain BL21(DE3). The expression vector carrying an insert of the TaeMCAII sequence as a template and oligonucleotide primers with the appropriate Cys replaced by Ala were used in the PCR reactions. The following pairs of mutagenic primers were designed (mutagenic bases in bold): 5′-GTCTCCGACTCA**GCG**CACAGTGGTGGC-3′ and 5′-GCCACCACTGTG**CGC**TGAGTCGGAGAC-3′ for the TaeMCAIIC140A mutant, and 5′-GCCGAGCTCAAGGGA**GCG**CACAACGACGTTGAC-3′ and 5′-GTCAACGTCGTTGTG**CGC**TCCCTTGAGCTCGGC-3′ for the TaeMCAIIC23A mutant. The PCR reaction with Pfu Turbo Polymerase (Stratagene, La Jolla, CA) was performed under the following conditions: 95 °C for 30 s, 16 cycles of 30 s at 95 °C, 1 min at 55 °C and 5 min 30 s at 68 °C. Before the transformation of competent *E. coli* DH5α cells, the PCR products were treated with* Dpn*I for 1 h at 37°C to digest the parental DNA. After the transformations, the plasmid vectors were isolated from single colonies and sequenced to confirm the mutations. The plasmid vectors with Cys140 or 23 replaced by Ala were used for the transformation of *E. coli* strain BL21(DE3), and the production of mutated proteins of TaeMCAII was induced by 1 mM IPTG. Cysteine mutants of TaeMCAII were expressed and purified as described above for wild-type TaeMCAII.

### Activity Assays of Wild-Type and Mutant TaeMCAII

The optimized buffer for wild-type and mutant TaeMCAII activity consisted of 100 mM HEPES (pH 7.0), 10 % glycerol, 10 mM DTT, 30 mM CaCl_2_ and 0.1 % CHAPS. The following buffer was used for determination of the pH profile of wild-type TaeMCAII: 50 mM acetic acid, 50 mM MES and 100 mM Tris. All assays were performed with 50 μM substrate and with 50 μg recombinant protein in 200 μl reaction mixtures using a colorimetric method. Metacaspase substrates, acetyl-valyl-arginyl-prolyl-arginyl-*p*-nitroanilide (Ac-VRPR-pNa) and acetyl-isoleucyl-arginyl-seryl-lysyl-p-nitroanilide (Ac-IRSK-pNa), were obtained from Biosyntan (Berlin, Germany), and caspase substrate acetyl-valyl-glutamyl-isoleucyl-aspartyl-p-nitroanilide (Ac-VEID-pNa) was purchased from Bachem (Bubendorf, Switzerland). The amount of hydrolyzed substrate was measured spectrophotometrically at 405 nm. For inhibition experiments, wild-type TaeMCAII was incubated with synthetic inhibitors, i.e., PMSF (1 mM), 1-trans-epoxysuccinylleucylamide-(4-guanido)-butane (E-64, 100 μM), chymostatin (100 μM), N^α^-tosyl-l-lysine-chloromethyl ketone (TLCK, 100 μM) and leupeptin (10 μM) for 1 h at 30°C prior to the addition of the substrate. All tested inhibitors were purchased from Sigma-Aldrich (St. Louis, MO).

### Structure Prediction and Sequence Analysis

**Fig. 1:**
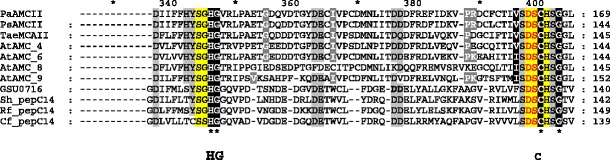
Multiple sequence alignment of plant type II metacaspases and selected bacterial metacaspase-like sequences. Conserved residues building active site (His87, Gly88 and Cys140) are the same in both groups of proteins are indicated. Sequences: plant metacaspases:* PaAMCII* *Picea abies* type II metacaspase,* PsAMCII* *Pinus silvestris* type II metacaspase ,* TaeMCAII* *Triticum aestivum* type II metacaspase (ACY82389),* AtAMC_X* *Arabidopsis thaliana* type I and II metacaspases, Bacterial sequences:* GSU0718* *Geobacter sulfurreducens* hypothetical protein;* Sh-pepC14* *Streptomyces himastatinicus*, peptidase C14 caspase catalytic subunit;* Rf_pepC14* *Rhodoferax ferrireducens* peptidase C14 caspase catalytic subunit;* Cf_pepC14* *Cellulomonas fini*, peptidase C14 caspase catalytic subunit

Multiple sequence alignment of eight plant metacaspases and four bacterial sequences possessing the C14 caspase catalytic subunit (Fig. 1) were constructed using the MUSCLE algorithm and were adjusted manually. Structure prediction for plant metacaspases was performed using the Fold Prediction Metaserver (http://genesilico.pl/), which provides access to 13 different fold recognition servers (Kurowski and Bujnicki 2003). Secondary structure predictions were obtained using a Metaserver consensus, which is constructed based on 16 secondary structure predictions methods (pssfinder, netsurfp, sspred, sspro4, spine, cdm, psipred, fdm, ssp, jnet, sspal, soprano, sable, prof, nnssp and gor).

### Structural Models of TaeMCAII

Sequence alignments between TaeMCAII, human caspase 7 (PDB 1F1J) and *Geobacter sulfurreducens* unknown protein GSU0718 were produced by the FFAS03 structure prediction method (Rychlewski et al. 2000; Jaroszewski et al. 2005) and were adjusted manually to accommodate the predicted secondary structures. Three-dimensional structure models were constructed using the program MODELLER (Sali and Blundell 1993; Eswar et al. 2006) with the combined Modeller9v8 Python scripts (automodel mode), model-multichain and model-ligand. Out of the models that were presented by MODELLER, the one with the most favorable *molpdf* score was selected for further analysis. The MetaMQAP server (Pawłowski et al. 2008) was used to estimate the correctness of the 3D models using a number of model quality assessment methods in a meta-analysis.

### Bioinformatics Identification of Potential Peptide Ligands for Modeled Structure of TaeMCAII

To choose the best candidates for in vitro activity studies, an in silico docking analysis was performed. The analysis of binding energies for potential ligands to the caspase-like domain of *T. aestivum* type II metacaspase model based on bacterial protein GSU0718 was accomplished using Glide 4.5 (Schrodinger®) to automate a procedure for ligand-receptor docking in standard and extra-precision mode. Glide (Grid-based Ligand Docking with Energetics) searches for favorable interactions between the receptor (the protein) and the molecules that were selected as ligands (i.e., the oligopeptides presented in Table 1). After generating a number of possible ligand orientations, the program evaluated the interactions of each of them with the receptor. The best conformations were allowed to progress to the final step of the algorithm, which was an energy minimization of the ligand–receptor complex involving the OPLS-AA force field energy grid. The final scoring using the Glide Score function was performed on energy-minimized poses. The ligand positions were then ranked using Glide Score values. The receptor structure was held rigid, and ligand structures were fully flexible.

**Table 1:** The list of oligopeptides as caspase and metacaspase ligands tested using bioinformatics tools

Caspase ligand	Metacaspase ligands	
valyl-glutamyl-izoleucyl-aspartate (VEID)	izoleucyl-arginyl-methionyl-arginine (IRMR)	izoleucyl-arginyl-prolyl-arginine (IRPR)
tyrosyl-valyl-alanyl-aspartate (YVAD)	valyl-arginyl-methionyl-arginine (VRMR)	glycyl-arginyl-arginine (GRR)
aspartyl-glutamyl-valyl-glutamate (DEVD)	izoleucyl-arginyl-seryl-lysine (IRSK)	izoleucyl-leucyl-threonyl-lysine (ILTK)
	izoleucyl-izoleucyl-threonyl-lysine (IITK)	izoleucyl-izoleucyl-seryl-lysine (IISK)

The set of potential caspase and metacaspase ligands for testing were selected based on literature data (Vercammen et al. 2006) and were prepared starting from raw amino acids using Maestro tools and the MacroModel 9.5 conformational search algorithm. More than 500 candidate ligand structures were obtained, which were screened and docked in the active site (substrate binding groove) of the modeled structure of TaeMCAII. All docking poses were scored, and the best scoring poses for each oligopeptide were saved.

## Results

### Autoprocessing of TaeMCAII Depends on Cys140

**Fig. 2a,b:**
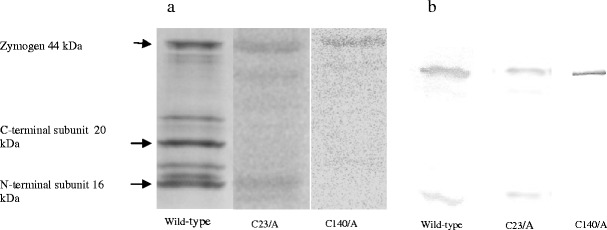
Autoprocessing pattern of wild type TaeMCAII and its two cysteine mutants (TaeMCAIIC23A and TaeMCAIIC140A.** a** SDS-PAGE and Coomassie Blue staining. Zymogen of wild-type TaeMCAII autoprocesses into two subunits.** b** Autoproccessing of wild-type TaeMCA II and its cysteine mutants detected by immunoblotting with anti-His antibody

The overproduction of TaeMCAII in bacterial cells led to its autoactivation, which consisted of the removal of a 66-amino-acid fragment with a molecular weight of 7.45 kDa from the middle of the zymogen. MS/MS analysis allowed us to identify the two autoprocessing sites of TaeMCAII as the C-terminal sites of Lys148 and Arg214. Autocleavage of the TaeMCAII zymogen resulted in the separation of N- and C-terminal subunits with molecular masses of 16 kDa and 20 kDa, respectively (Fig. 2a). When His_6_ tag-purified wild-type TaeMCAII and mutant form TaeMCAIIC23A were analyzed by PAGE and immunoblotted with anti-His antibodies, the appropriate fragments corresponding to those two subunits and zymogen were visible (Fig. 2a,b). In contrast, only the zymogenic form of metacaspase was visible for mutant TaeMCAIIC140A, and the fragments with molecular masses corresponding to the two subunits of active enzyme were not detected (Fig. 2a,b).

TaeMCAII preferentially cleaves peptide bonds after arginine and to a lesser extent after lysine at the P1 position of the substrate.

**Fig. 3:**
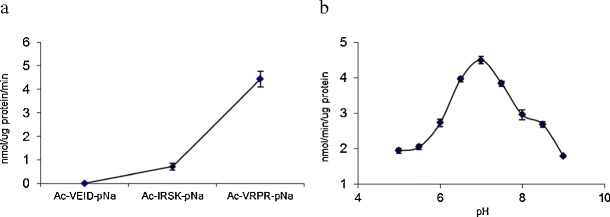
**a** Substrate specificity of TaeMCAII. Three peptide substrates were chosen on the basis of docking results. Mean values are shown with standard deviation calculated from three replicates. **b** Proteolytic activity of TaeMCAII against Ac-VRPR-pNa substrate at different pH. Mean values of three replicates are presented

**Fig. 4:**
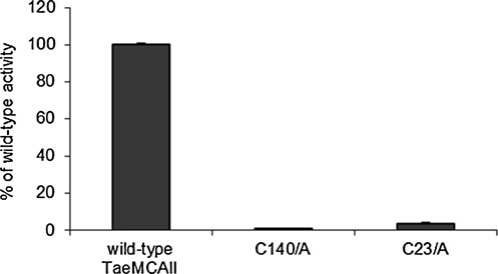
Influence of **a** calcium ions and **b** different protease inhibitors on TaeMCAII substrate hydrolysis and activity, respectively. Mean values and percent activity, under optimal conditions (Ac-VRPR-pNa as a substrate, pH 7, and 30 mM Ca^+2^ concentration), of three replications are presented

Three different synthetic substrates with p-nitroaniline at the C-terminus were chosen to study their hydrolysis by TaeMCAII using bioinformatics tools. One of these substrates, Ac-VEID-pNa, is known to be an optimal substrate for caspase-6. Two others, Ac-VRPR-pNa and Ac-IRSK-pNa, have been used in earlier studies as specific substrates of AtMC9. No TaeMCAII activity was observed against the caspase-6 substrate Ac-VEID-pNa (Fig. 3a). TaeMCAII appeared to be an arginine-specific enzyme, as it hydrolyzed Ac-VRPR-pNa with the highest efficiency (Fig. 3a). For the substrate with lysine at the P1 position, AC-IRSK-pNa, TaeMCAII exhibited six-fold lower activity than for Ac-VRPR-pNa (Fig. 3a). Hydrolysis of Ac-VRPR-pNa was detectable in the pH range of 5.0–9.0, with highest activity at neutral pH (Fig. 3b). To investigate the importance of the two catalytic cysteines 140 and 23 with regard to the activity of TaeMCAII, two wheat metacaspase mutants, TaeMCAIIC140A and TaeMCAIIC23A, were tested for the efficiency of Ac-VRPR-pNa hydrolysis. In the case of TaeMCAIIC140A, hydrolysis was completely abolished (Fig. 4). The latter mutant, TaeMCAIIC23A, had very little ability to cleave the substrate and exhibited only 3.5 % of wild-type activity (Fig. 4).

### Influence of Protease Inhibitors on TaeMCAII Activity

**Fig. 5:**
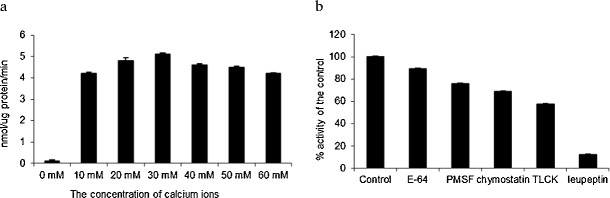
Influence of **a** calcium ions and **b** different protease inhibitors on TaeMCAII substrate hydrolysis and activity, respectively. Mean values and percent activity, under optimal conditions (Ac-VRPR-pNa as a substrate, pH 7, and 30 mM Ca^+2^ concentration), of three replications are presented

The effect of several protease inhibitors with Ac-VRPR-pNa as a substrate was assessed. Incubation of TaeMCAII with an irreversible inhibitor of serine and cysteine proteases, PMSF, (1 mM) and with irreversible inhibitor E-64 influenced TaeMCAII activity to a minor extent, as the enzyme retained 76 % and 89 % of its activity, respectively (Fig. 5b). The activity of wheat metacaspase was considerably affected by chymostatin, an inhibitor of chymotrypsin and lysosomal cysteine proteinases, and TLCK, an irreversible trypsin inhibitor (Fig. 5b). The activity of TaeMCAII decreased by over 30 % following incubation with chymostatin and by 43 % after incubation with TLCK. The greatest influence on TaeMCAII was seen with the arginyl competitive protease inhibitor leupeptin, which decreased its activity significantly by 88 % (Fig. 5b).

### TaeMCAII Requires a High Concentration of Ca^+2^ for Activity

Millimolar ranges of Ca^+2^ concentrations were required for TaeMCAII to cleave its optimal peptide substrate, Ac-VRPR-pNa. Without calcium, TaeMCAII exhibited only 2 % of its optimal activity, which was reached in the presence of 30 mM Ca^+2^. A slight decrease in substrate hydrolysis by TaeMCAII was observed when this optimal calcium level was exceeded (Fig. 5a).

### Alignment Analysis

**Fig. 6:**
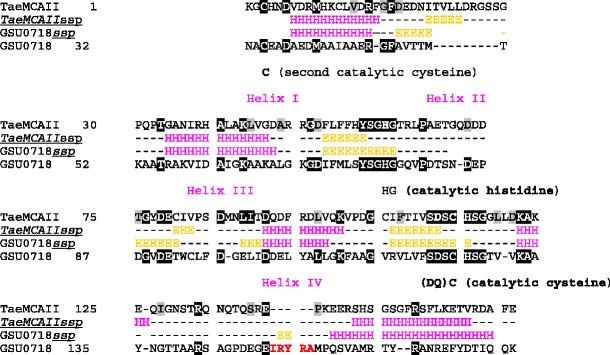
Alignment of *T. aestivum* type II metacaspase (TaeMCAII) amino acid sequence (the GenBank accession number: ACY82389) and* G. sulfurreducens* unannotated protein GSU0718 (FASTA sequence of 3BIJ.pdb file—chain A).* TaeMCAIIssp* Consensus for TaeMCAII sequence secondary structure predictions obtained using 16 methods: pssfinder, netsurfp, sspred, sspro4, spine, cdm, psipred, fdm, ssp, jnet, sspal, soprano, sable, prof, nnssp and gor.* GSU0716ssp* Secondary structures assignments from PDB files

Following bioinformatics analysis of the TaeMCAII sequence and after running regular PSI-BLAST searches on the nrNCBI database, a multi-alignment of seven plant type II metacaspases and four bacterial metacaspase sequences was constructed (Fig. 1), presenting a region that contains the catalytic Cys/His dyad and its neighboring residues. Based on the sequences, a potential template—a homologue with a known structure, bacterial GSU0716—was chosen for further analysis using the FFAS03 algorithm. The results of the profile-profile alignment of TaeMCAII and the above-mentioned sequence are presented in Fig. 6, together with the predictions of secondary structure elements for TaeMCAII (consensus obtained after running 16 different predictor algorithms) and ss annotations that were derived from PDB files in the case of GSU0716 (3BIJ).

### Structure Modeling and Analysis

Determining the appropriate template is crucial to obtaining accurate data from protein modeling. Plant metacaspases show rather moderate sequence similarity to animal caspases, and only for caspases can one find experimentally resolved structures in the Protein DataBank. However, poor similarity did not disturb the construction of plant metacaspase structural models based on human caspase-7 templates (Belenghi et al. 2007).

Attempting to obtain an accurate model of the newly sequenced *Triticum aestivum* type II metacaspase, we also started with the caspase-7 template, but decided to select another structure as the modeling template after running fold predictions methods. The chosen PDB record contained the structure of an unknown *Geobacter sulfurreducens* protein, described as GSU0716 (Northeast Structural Genomics target GsR13). This was indicated by almost all available fold prediction algorithms, including COMA, COMPASS, Phyre, FFAS03 and HHsearch, as the best scoring hit for the TaeMCAII sequence. Moreover, unknown protein GSU0716 has close homologs within the group of the bacterial members of the peptidase_C14 pfam00656 family and shows homology to plant metacaspases with e-values in the range of 7e−10 to 2e−09. Even a preliminary BLAST search followed by hmmpfam and HHsearch analysis revealed the presence of Pfam Peptidase C1 domain PF00656, which is called the caspase domain. After analysis of the profile–profile alignments of TaeMCAII and GSU0716 (based on the results of the FFAS03 method) (Fig. 6), we observed two highly conserved regions between TaeMCAII and GSU0716 near the catalytic dyad His/Cys (5- and 7-amino-acid-long motifs: YSGHG and SDSCHSG), which are also present in another plant metacaspases and aligned bacterial metacaspase-like sequences (Fig. 1), in addition to high conformity of the secondary structure assignments and predictions for the TaeMCAII and GSU0716 sequences.

However, the lack of exposure of key catalytic residues in the hypothetical binding groove could discourage the use of the 3BIJ structure as a metacaspase-like modeling template. Looking closer at the molecular surface of the GSU0716 putative substrate binding, we noticed that four presumably catalytic residues (Cys135, His84, Gly85 and Asp133 in GSU0716 numbering) were hidden or were not sufficiently exposed to make contact with the potential substrate. In the original GSU0716 structure, only His84 from the putative catalytic dyad His-Cys was partially exposed. The catalytic cleft was blocked by a short oligopeptide, IRYRA, that covered the surfaces of Cys135, Gly85 and Asp133 residues. However, after removing this fragment, the GSU0716 catalytic residues in the binding groove were exposed to make contact with the potential substrate.

**Fig. 7:**
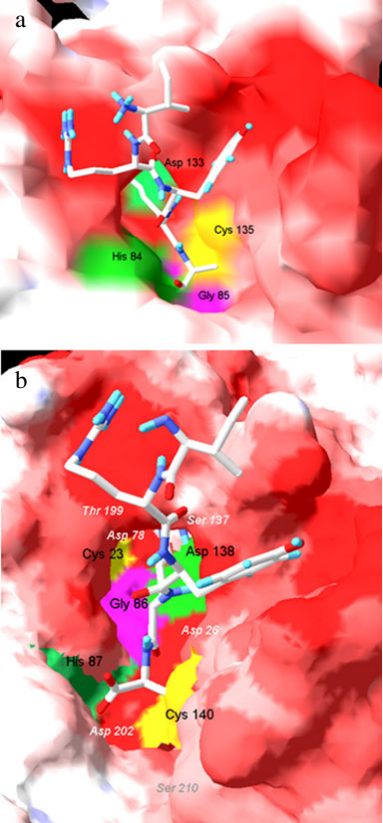
**a** Electrostatic potential map (EPM) of the surface of bacterial protein GSU0716 structure, template considered for TaeMCAII modelling, with the IRYRA peptide (part of the molecule identified to be a possible co-crystalized substrate) superimposed. **b** EPM of modelled TaeMCAII structure with IRYRA peptide in the binding groove. The putative substrate is presented in stick molecular representation in CPK color to show the surface of binding grooves. Key residues are depicted in surface representation and distinguished by different colors. Negatively charged residues in the neighborhood of the binding site are labelled in* white*

To investigate a potential template for *T. aestivum* type II metacaspase modeling, an analysis of the electrostatic potential of the surroundings of the putative substrate binding site was performed (Fig. 7b). Figure 7a shows the exposed putative ligand binding groove of GSU0716 after removing the IRYRA peptide, which was crystallized as part of the 3BIJ structure. The electrostatic potentials of the residues near the GSU0716 putative active site were slightly negative, and this characteristic aided in determining the substrate specificity, as the IRYRA oligopeptide is enriched in amino acids with positively charged side chains.

**Fig. 8:**
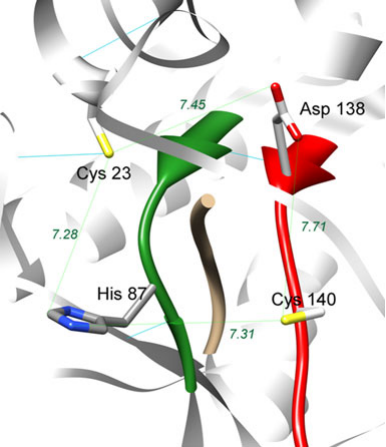
Model of *T. aestivum* type II metacaspase active site 3D structure constructed based on the GSU0716 template. Probable location of the ligand (depicted in* ribbon representation*) is approximated by superposition of GSU0716 IRYRA peptide (*orange*). Backbones of conserved YSGHG and SDSCHSG sequences are presented as* red* and* green ribbons*, respectively. Residues suspected of catalytic functions (in cysteine protease hypothesis) are His 87 and Cys 140. Residues Asp 138 and Cys 23 mentioned in the discussion are depicted in stick representation

The hypothetical conformation of the TaeMCAII active site model based on the GSU0716 structure is shown in Fig. 8. The putative ligand position is indicated by the superimposition of the IRYRA peptide from the template structure. Four important and conserved residues according to MSA from Fig. 1, including the putative catalytic dyad, are depicted in all-atom representation, and the model obtained is discussed below.

The obtained Glide Scores (the general measure used for the evaluation of a given ligand pose in receptor binding sites) for the set of oligopeptides that were suspected to bind to the type II metacaspase active site indicated that the best potential ligands for the TaeMCAII enzyme were oligopeptides VRPR and IRSK, as these peptides resulted in the highest scores.

## Discussion

Very few plant metacaspases have been identified and well characterized (Vercammen et al. 2004, 2006; Bozhkov et al. 2005; Sunström et al 2009). This study describes the biochemical characterization and the results of homology modeling of a cereal type II metacaspase from wheat (*Triticum aestivum*), TaeMCAII. Modeling was based on a bacterial template, as there are no experimentally resolved tertiary structures of plant metacaspases in the Protein Databank. The reasons for choosing this specific template are described in the Results section.

Members of the CD clan of proteases, including metacaspases, are known to recognize the residues at the N-terminal side of the scissile bond, named the P1 residue, in their substrates (Tsiatsiani et al. 2011). Metacaspases, like caspases, recognize three or more amino acids residues N-terminal to the P1 position. This position is acidic in the case of caspase substrates and is basic for metacaspase substrates (Earnshaw et al. 1999). More in vitro evidence was provided here in that metacaspases cleave their substrates preferentially after the basic amino acids Arg or Lys (Fig. 3a). Like other known metacaspases, e.g., AtMC9 and mcIIPa, TaeMCAII exhibits much greater activity towards substrates that contain Arg at the P1 position than Lys (Fig. 3a). According to the homology model, which was based on bacterial template GSU0716, negatively charged residues (Asp138, Ser137, Asp26, Asp202, Thr199, Ser210, Asp78 and Ser215) near the TaeMCAII active site can facilitate the binding of positively charged ligands, i.e., peptides enriched in Arg or Lys (Fig. 7b). The substrate specificity of TaeMCAII towards Arg in the P1 position was also confirmed by inhibition studies. TaeMCAII activity was blocked completely by an arginyl inhibitor, leupeptin, and TLCK and chymostatin also sufficiently inhibited its activity (Fig. 5b). Nevertheless, MS/MS analysis revealed the autoprocessing sites of TaeMCAII as the C-terminal sides of Lys 148 and Arg 214, removing a linker region of 7,457 Da and separating two caspase-like subunits (Fig. 2a, b).

Up to now, all well-characterized metacaspases have had larger N-terminal subunits than C-terminal subunits, and correspond to p-20 and p-10 caspase subunits, respectively (Vercammen et al. 2004; Bozhkov et al. 2005). In contrast, the TaeMCAII N-terminal subunit was smaller than the C-terminal subunit (Fig. 2a). It should be noted that, similar to caspases and other known metacaspases, the catalytic dyad Cys140 and His87 of TaeMCAII are located in the N-terminal subunit (Fig. 6). The overproduction of TaeMCAII in *E. coli* resulted in its concomitant autoprocessing depending on the catalytic Cys140. This autoprocessing was completely abolished in the TaeMCAIIC140A mutant (Fig. 2a,b). This mutant form of TaeMCAII was also found to be inactive (Fig. 4). Processing is necessary for the activation of the zymogens of all known type II metacaspases and executioner caspases of animals (Tsiatsiani et al. 2011). Multi-sequence alignment (MSA) and a homology model confirmed the significance of Cys140 in the TaeMCAII active site (Figs. 1, 8). In bacterial and plant metacaspases, including TaeMCAII, three residues before the catalytic histidine (Tyr, Ser and Gly) and six residues surrounding the catalytic Cys140 (Ser, Asp, Ser and His, Ser and Gly) are strictly conserved (Fig. 1). These conserved motifs appeared to be specific only for plant metacaspases and bacterial metacaspase-like sequences, but are not observed in animal caspases (data not shown). MSA of the amino acid sequences of known plant metacaspases and TaeMCAII revealed the existence of a second highly conserved cysteine residue (Cys23 in TaeMCAII ) that is involved in catalytic process in metacaspases (Fig. 1). The obtained homology model of TaeMCAII based on the bacterial putative metacaspase-like protein helped explain the role of Cys23 in the catalytic process (Fig. 7b). According to this model, two conserved cysteines near the catalytic His87, Cys23 and Cys140 may participate in the nucleophile attack. The distances between the His87 ring imidazole N atom and the Cys thiol groups, crucial for the reaction mechanism, are 7.31 Å (Cys140) and 7.28 Å (Cys23). Both cysteines can donate a proton to the histidine ring (Fig. 8). Docking results show that the peptide substrate was bound in a position that enables both Cys23 and 140 to exchange a proton (Fig. 8).

As revealed by analysis of the TaeMCAIIC23A mutant, Cys23 is not required for activation of the wheat metacaspase, but is needed for its activity. The proper autoprocessing was found in the TaeMCAIIC23A mutant, but it exhibited very low activity compared to the wild-type protein (Figs. 2, 4). The implied functions of two corresponding catalytic Cys residues during autoprocessing and enzyme activity were also confirmed during the studies with AtMC9 (Belenghi et al. 2007). The biochemical studies and modeling results described here indicate the importance of both cysteines for the activity of TaeMCAII, but Cys140 is required for the autoactivation process.

On the basis of TaeMCAII’s neutral pH preference, one might suggest that TaeMCAII is a cytoplasmic proteinase (Fig. 3b). The majority of metacaspases that have been studied so far, including *Arabidopsis* metacaspases, (AtMC4 and AtMC9), *Picea abies* mcIIPa, *Leishmania* (LdMC1and LdMC2) and *Trypanosoma* (TbMCA2) metacaspases, require neutral or slightly basic pH for optimal activity in vitro (Vercammen et al. 2004; Bozhkov et al. 2005; Moss et al. 2007; Lee et al. 2007). To date, AtMC9 is the only known metacaspase that requires an acidic environment for its activation and action (Vercammen et al. 2004). Until now, an organelle localization was found only for *Arabidopsis* metacaspase AtMCP1b, and it was found in chloroplasts (Castillo-Olamendi et al. 2007). It would be useful to know in which organelles metacaspases are active in order to suggest their possible roles in signaling pathways in plant cells. It is interesting that TaeMCAII, similar to *Arabidopsis* and *Picea abies* metacaspases, had highest activity at millimolar levels of Ca^+2^ ions (Fig. 5a) (Vercammen et al. 2004; Bozhkov et al. 2005). Recently, it was demonstrated that the AtMC4 autoproteolytic process is Ca^+2^-dependent (Watanabe and Lam 2011). It should be noted that under many stress conditions, e.g., salinity, cold and heat stress, the concentration of cytosolic calcium increases dramatically (Xiong et al. 2002; Kang et al. 2011). It is also known that high concentrations of calcium ions is toxic for plant organelles; thus, there are some specific channels/pumps that regulate the movement of Ca^+2^ in and out of cells and organelles (Xiong et al. 2006). Some plant organelles are more tolerant of high levels of this ion than others. The highest concentration of Ca^+2^ ions, exceeding 50 mM, is found in the endoplasmic reticulum (ER). Plastids, mitochondria and vacuoles contain millimolar levels of Ca^+2^ but much less than the ER (Hepler 2005). The role of calcium ions in stress signaling in plants and in plant PCD has been demonstrated previously (Xiong et al. 2006; Hepler 2005). It should be noted that the very high levels of Ca^+2^ ions required for optimal activity of the wheat metacaspase TaeMCAII and other plant metacaspases, e.g., AtMC4 and mcIIPa, may play a significant role in the adaptation and acclimatization of these plants to stressed environmental conditions. The aim of our future investigations will be the localization of TaeMCAII in subcellular organelles and in vivo measurements of its catalytic activity under various stress conditions, followed by elevated cellular Ca^+2^ concentrations. The results should be highly relevant to agriculture, as they indicate possible signaling pathways that influence the acclimation of cereal plants to stress conditions.
